# Observations on the Department of Pathology and Pathological Anatomy

**Published:** 1843-10

**Authors:** 


### ART. IX.

1. *Beobachtungen auf dem Gebiete der Pathologie und Pathologischen Anatomie, gesammelt von* Dr. JOH. FRIED. HERM. ALBERS, Professor der Medezin, &c. in Bonn. Dritter Theil.—*Bonn*, 1840. 8vo, pp. 195.

*Observations on the Department of Pathology and Pathological Anatomy, collected by* Dr. J. F. H. ALBERS, Professor of Medicine, &c. at Bonn. Third Part.—*Bonn*, 1840.

2. *Waarnemingen in het Gebied der Pathologie an der Pathologische-Anatomie.* Door Dr. J. F. KERST, Chir. Mazoor, &c.—*Utrecht*, 1839. 8vo, pp. 214.

*Observations in the Department of Pathology and of Pathological Anatomy.* By Dr. J. F. KERST, Surgeon-major, &c.—*Utrecht*, 1839.

3. *Bijdrage tot de Ontleedkundige Ziektekennis.* Van F. S. ALEXANDER, Med. Doct. en Prof. to Utrecht.—*Gorinchem*, 1835. 8vo, pp. 38.

*Contributions to Anatomical Pathology.* By F. S. ALEXANDER, M.D., and Professor of Medicine at Utrecht.—*Gorinchem*, 1835.

I. THE first part of Professor Albers' work was noticed in our Fifth Volume, and we have often, both before and since, had occasion to speak of his writings.* Yet there has seldom been reason to praise the results of his labours; for though there is much accuracy in his descriptions of what he actually sees, yet there is a want of discernment of the differences in the things which he investigates, and an unwarrantable freedom in generalizing from a few facts, which entirely neutralize his claim to merit. The present series of essays clearly demonstrate both these faults.

The first essay is "*on the simple ulcer of the stomach,*" which commonly terminates by perforation of the walls of that organ, and fatal peritonitis. On the morbid anatomy of the disease nothing is said beyond what is generally known. The author enters at some length into the diagnosis of its early progress; and his remarks might have been quoted, had he not seemed so much more definite than he is warranted in being by the evidence before him, as to excite suspicion that he speaks rather from what the morbid anatomy of the disease suggests of its progress

* See vol. II. p. 535; vol. III. pp. 220, 246; vol. VIII. p. 544; vol. IX. p. 445.

during life, than from actual observation of the signs of its course. On the experience of only five cases he describes the disease as passing with well-marked characters through several distinct stages; and omits to notice, as if he were ignorant of the fact, that its existence is often not testified by any symptom whatever, till the perforation takes place.

The second paper is "*on somnambulism*, and will be noticed elsewhere. The third is "*on dilatation of the bronchi forming cavities*;" but it contains nothing with which English readers are not familiar. The fourth relates to "*encysted tumours in the perichondrium of the thyroid cartilage.*" It contains a description of two preparations, in each of which that part was occupied by a tumour nearly an inch long. In one the tumour was composed of a fibrous tissue in concentric layers inclosed in a dense fibrous capsule; in the other it consisted of a cyst half a line thick, containing a cellulo-fibrous, and a granular, pultaceous substance. The author compares these growths with one of a similar kind found within the larynx, and described in the first part of his work.

The fifth essay relates to a case in which a fluid containing fibrine, and separating after its removal into serum, and a coagulum was drawn from the interior of the abdomen. But there is no evidence to show what was its source.

The title of the last and longest essay is "*on hyperostosis, osteoporosis, and exostosis.*" Here, again, we think it unadvisable to notice the author's account of the signs of the diseases he describes; for there is no reason to believe that he observed them during life. On the contrary, he acknowledges that he is acquainted with the appearances of the diseased parts only through the old preparations in the Bonn Museum, and a few examinations made soon after death. Perhaps too his morbid anatomy might as summarily be passed over; but in pointing out his errors, some useful truth may be told.

His primary division of hyperostosis is into hypertrophy of the medulla, or *osteomyelosis*, and hypertrophy of the compact tissue of bone, or *hyperostosis* properly so called.

In his account of the first he confounds two distinct affections; rickets, and atrophy of bone. He speaks of the excessive fat being modified so as to be of a "dark dirty red, yellow, or even ash-gray" colour; and says he found it thus in the interior of the femur of a rickety child. Now the material here described is not fat, but a peculiar gelatinous substance which occupies large spaces formed in the cancellous tissue of rickety bones, and in its general aspect resembles soft provisional callus. This therefore is not hypertrophy of the medulla, nor does it bear any relation to that which is rather less improperly so called. Of this the author says, that the medulla either is very consistent and exactly fills all the cavities, (which, we think, is only what is healthy in a fat man;) or, "the wall of the bone has at the same time diminished, and the osseous lamellæ which form the cells of the medullary membrane, are become thinner and have decreased in numbers. I could," he adds, "in one case remove the medulla out of the cavity without any trouble whatever, and I found only a few lamellæ forming cells; yet the femur belonged to an adult man, who died apoplectic." The description is very accurate, but the change is no hypertrophy; it is the usual condition of bones which have been atrophied through disuse, either because the patient has been bed-ridden, or (when the change affects only certain bones,) because, in consequence of paralysis, a stiff joint, or some such condition, the muscles have long ceased to move them. In short, the "hypertrophy of the medulla" of Professor Albers, is ordinary atrophy of bone. The patient in whom he found it had, without doubt, been hemiplegic long before his fatal attack of apoplexy.

Hypertrophy of the osseous substance, or hyperostosis properly so called, is divided into four kinds; 1, Simple hyperostosis of the bone; 2, Hyperostosis with a morbid alteration of tissue; 3, Hyperostosis with coincident new formation of bone on the outer surface; 4, Local hyperostosis, exostosis and osteophyte.

Nothing can show better the impropriety of making morbid anatomy the basis of pathology. The author describes the distinctive signs of each of these states of disease; yet the most common condition is that in which the first three exist together. The majority of the bones that have been affected with rheumatism are at once thicker and denser than usual, distinctly altered in their tissue, and beset with growths of bone from their outer surfaces. So are those near which ulcers of the integuments have long existed. And, on the other hand, the state defined in No. 1 may be the result of several different circumstances; for instance, of the natural growth of an excessively used bone, of the growth of bones, such as those of the skull, which sometimes increase as the volume of the organs which they contain decreases, and of simple chronic inflammation. The author's description of these three forms of disease might therefore be merged in one: or rather they should be arranged so as to illustrate each of the several processes of which they are the results.

It would be unprofitable to analyse all that is said of thickened bones, and we will therefore only report what is said of their microscopic structure. It is, that the corpuscles in bone which is merely thickened, are smaller than in normal bone, and that the calcigerous canals are fewer and more minute, and in some parts even entirely absent. The osseous substance moreover is thickly scattered with black spots formed by a deposition of pigment, and varying in size up to that of a pin's head. He believes therefore that the hypertrophy consists, not in an actual growth of bone, but in a mere addition to the earthy matter. On consulting the drawing which is appended to the work, we find, however, reason to doubt this conclusion; for it exhibits the corpuscles as large, and the canals as numerous as they are in the majority of sections of bone, and, if we are not much mistaken, the spots of pigment, as the author considers them, are oblique sections of Haversian canals. They have just the same form, size, and direction, as those canals: they branch like them, and have the same general appearance; and they resemble no kind of pigment whatever. The author's observations therefore do not seem to warrant the conclusions, which is opposed to the results obtained by others, that the addition to bones when they are merely hypertrophied is anything but normal osseous tissue.

In the bones that are thickened with considerable alteration in their obvious structures, the case is different. The new bony substance shows no trace of corpuscles or canals, but an irregular appearance, as of longitudinal fibres with scattered granular dark bodies.

Of exostosis, or partial hyperostosis, the author describes three kinds, namely, ivory exostosis, cellular exostosis, and exostosis formed by the expansion of one side of the medulla. But we cannot find that he adds anything important to that which is contained in many works on the anatomy of tumours, and of diseased bones.

II. Dr. Kerst's observations are of more modest pretensions. They consist of nineteen cases illustrative of morbid anatomy, and require no further comment than that they are clearly and honestly related. We shall make abstracts of those which are most interesting.

The first case relates to the examination of the eye of a dog which had been acutely inflamed in consequence of severe external injury a year and a half before death. The cornea was flattened and completely opaque; and formed the base of the cone-shaped body into which the globe had shrunken. Immediately behind the cornea there was an elevated fibro-cartilaginous ring, about four lines broad, and going all round the globe; it was produced by a thickening of the sclerotica, which was here from a line and a half to two lines thick. Behind it the globe felt empty; there was no trace of hyaloid membrane or vitreous humour, but, in their place a small quantity of sanious fluid. The posterior part of the retina was very thin, and had a finely-granular surface; the anterior was thickened; it was everywhere inseparably adherent to the choroid, which was itself healthy. At the posterior surface of the lens there was a tough and very vascular membrane firmly attached by its margins to all the adjacent parts; the capsule was thickened and wrinkled. The iris was converted into a black shapeless mass, which filled the posterior chamber; it did not adhere to the capsule, but, in front, it was closely adherent to the cornea, and, at the situation where the pupil should have been, dipped into its substance. The optic nerve seemed healthy. (Kerst, p. 11.)

The fourth observation of an "excessively large fibrous tumour taken for an aneurism" has the more interest from the error having been committed by Dr. Engelhardt, one of the most skilful surgeons in Holland. The patient was a soldier, nineteen years old, who said that about three weeks before his admission, as he was walking, he felt a sudden pain in the left ham, and on reaching home found a swelling there, which in twenty-four hours increased to the size which it presented on his arrival at the infirmary. At this time the left knee was much enlarged, but the pain had not increased. There was a swelling as large as a goose's egg in the ham, partly compact, partly fluctuating, and, on moderate pressure, distinctly pulsating. In the notion that it was a false aneurism compression was employed for some time; but as the swelling increased, and œdema of the leg came on, the femoral artery was tied. (Two ligatures were applied and the vessel was divided between them; a plan which Dutch surgeons are perhaps not aware that the countrymen of the Hunter whom they so admire, have long since abandoned.) After the operation, however, which in itself was successful, the tumour continued to grow as rapidly as before; the patient began to cough as if from organic disease of the lungs, and gradually sunk.

On examining the body the internal organs were found healthy, except the right lung, which was completely converted into a brain-like mass. The tumour in the ham was fifteen inches long and twenty-four inches in circumference. It was pear-shaped, and lobed, and adhered firmly to the periosteum of the femur and tibia. On its surface were five sacculi, holding between five and six ounces of a sero-sanguinolent fluid, and apparently formed by the separation of the two layers, of which a tough fibrous membrane, enveloping the whole tumour, was composed. The tumour itself consisted, in part, of a dry fibrous reticulated tissue, divided by partitions of looser tissue into lobes, and separated into two chief portions by a plate of bone; and, in part, of a softer substance, easily broken, rose coloured, interspersed with fibrous tissue, and containing many cells filled with serous fluid. The femur and tibia were healthy. So also was the joint, except that there was a small mass of soft substance, like that just described, on the inner surface of the synovial membrane. The artery ran over the tumour, and between two of the sacs of fluid on its surface: and through these its pulsation had been so communicated as to give the sensation of the whole tumour pulsating. (Kerst, p. 27.)

The next interesting case is one of tubercles in the heart. The patient was twenty-one years old, and died excessively emaciated, but without any symptom that excited suspicion of disease of the heart. He had crude tubercles at the upper part of the left lung, and both mediastina were filled by enlarged and tuberculous lymphatic glands. The heart was of ordinary size, and was everywhere as closely adherent to the pericardium as if the latter had been its outer membrane. Between the two, however, there were masses of tubercle, some as large as a hazelnut. At the upper part of the outer wall on the right side, there was, near the surface of the muscular tissue, a tubercle as large as a pea, and at the apex one much larger. All the tubercles had gone into the stage of softening. The muscular substance around them exhibited no morbid change, and the vessels were unaltered; but there was yellow-softening of the outer part of the wall of the left ventricle; a layer of muscular substance, thus diseased, lying between the healthy external and internal layers.

The ninth and tenth cases relate to tuberculous matter occurring in the vertebræ. They are detailed as very rare occurrences, and the author says, that before meeting with them he had sought in vain for examples of the disease. Tilanus also, one of the most experienced surgical pathologists in Holland, in his "Schets der Heelkunde," makes no mention of tuberculous disease in bones. These facts are of much importance, for since scrofula in all its other forms is abundant in Holland, they add to the evidence that the French pathologists and their followers in this country are in error, when they speak of tuberculization as an ordinary change in the osseous tissue. It is one of the rarest.

The eleventh case illustrates one of the most serious, and not an unfrequent result of operations on the testicle. A man, aged forty-one, to all appearance of a healthy constitution, had enlarged testicles. The disease had existed for a year, and they had both degenerated into a kind of medullary substance. They were both removed at once; and in the evening after the operation the patient began to complain of pain and tenderness of the abdomen, which rapidly increased, assumed all the characters of acute peritonitis, with scarcely any appearance of inflammation externally, and proved fatal on the fourth day. At the examination all the thoracic and abdominal viscera were found healthy; but the whole of the superficial fascia and the subcutaneous tissue of the walls of the abdomen and the sides of the trunk were converted into a grayish-black soft *putrilage*, mixed here and there with pus. The degeneration extended from the scrotum to the axillæ and the scapulæ; but near the latter had not reached so extreme a stage. The spermatic cords and the inguinal canals were healthy.

Shortly after this case similar symptoms occurred in a man who had been tapped for hydrocele, and from whom a portion of the tunica vaginalis had been excised. Suspecting from his experience in the preceding case what had happened, Dr. Kerst at once made free incisions through the integuments of the abdomen; large portions of dead cellular tissue were discharged through them, healthy suppuration was then established, and the patient recovered. (Kerst, p. 90.)

The rest of the observations are of less interest than those which we have abstracted: the last five are cases of fatal purulent phlebitis, after wounds and operations, but they are not sufficient to throw any fresh light on that obscure disease.

III. In Professor Alexander's pamphlet the first three cases contain post-mortem examinations of lunatics, in all of which he found "adhesion of the pia-mater to the surface of the brain; an adhesion which was in many parts so firm that the membrane could not be removed without tearing the substance of the brain." (p. 11.) He regards it as a sign, that in all these cases there had been inflammation of the adherent parts.

The eighth observation is a remarkable one of accumulation of fat in the thorax and abdomen. The patient was a man forty years old, who had lived freely and suffered much from dyspepsia. For a long time before his death he had complained of pain and oppression in his chest, which had been variously treated without success: and to these succeeded shortness of breath, so that he was often obliged to stop when he was walking. No distinct disease, however, could be made out, but he grew worse and the oppression in the chest disabled him from all occupation. Shortly before his death he was affected by catarrh, which greatly increased his dyspnea; he became feverish and excessively restless; he could not be kept in bed, his distress was so great, and, as he was sitting up, he died suddenly.

On examination, his body was found robust; but there was a want of correspondence in the limbs, and the shape of the trunk was more like that of a woman than that of a man. A large quantity of fat was accumulated in the integuments. The right lung was completely adherent; the left rather less so; the tissue of both was healthy. The anterior mediastinum was filled, and the front of the diaphragm was thickly covered by fat of various consistence. The pericardium was covered by two lumps of fat of such a size that they must have impeded the heart's action. The heart itself was very fat, and its vessels were congested, but it was not otherwise diseased. The abdomen seemed to be filled by one shapeless mass of fat of several pounds weight: the mesentery, mesocolon, and all the parts in which it is usually found in moderate quantity were overladen with the same substance. The liver was very large; the gall-bladder very small; but all the other abdominal organs were healthy. (Alexander, p. 30.)

After what was said of the feminine form of the trunk, it is to be regretted that the state of the genital organs was not noticed in this case. We remember examining a very similar case in which the accumulation of fat was referrible to degeneration of the testes.

The last observation is one of supposed perforation of the intestine by an ascaris: but, as in many cases of the kind, there is not sufficient evidence that the perforation was not spontaneous, and that the worm did not escape into the abdomen some time after it took place, or perhaps even after death. This view is the more probable, because the patient had phthisis; the aperture was in the middle of an ulcer, resembling a phthisical one, in the jejunum; and there was another ulcer of the same character in the ileum.

---

